# Anisosmotic Modulation of Mutant Huntingtin Aggregation vis-a-vis HSP70 Induction—Implications for Aging, Hypo-Hydration, and Neurodegeneration

**DOI:** 10.1021/acschemneuro.5c00966

**Published:** 2026-02-17

**Authors:** Alice Y. C. Liu, Kelvin Y. Kwan, Clarissa Kwan, Kuang Yu Chen

**Affiliations:** † Department of Cell Biology and Neuroscience, Rutgers State University of New Jersey, Nelson Biology Laboratory, 604 Allison Road, Piscataway, New Jersey 08854, United States; ‡ Department of Chemistry and Chemical Biology, Rutgers State University of New Jersey, Nelson Biology Laboratory, 604 Allison Road, Piscataway, New Jersey 08854, United States

**Keywords:** polyQ-Huntingtin reporter protein, HSP chaperone, aging, hypo-hydration, neurodegeneration

## Abstract

Suboptimal cell hydration is a significant risk factor for age-related deterioration and disease vulnerability. Herein, we use a Huntington disease cell model to evaluate osmolarity-dependent modulation of (1) aggregation of polyQ-expanded mutant Huntingtin-EGFP reporter protein as a readout for structurally dynamic disease proteins versus (2) induction of HSP70 chaperone to report on stress-induced lability of folded proteins. Cell impermeant alkali-metal salts and polyethylene glycols were added to cell media to osmotically dehydrate cells for crowding, whereas water was added to swell cells for macromolecular dispersion. Cell image and biochemical analyses show that addition of sodium chloride and other alkali-metal salts to cell media promoted aggregation of mHTT^Exon1^-EGFP protein into forming “inclusion bodies” (IBs) in live cells, while concurrently dampened the induction of HSP70 by heat shock. Conversely, a hypo-osmotic medium tempered the compaction of mHTT^Exon1^-EGFP into forming IBs while increasing the induction of HSP70. Cell impermeable PEGs likewise promoted mHTT^Exon1^-EGFP aggregation. These observations underscore the importance of an iso-osmotic cell environment for balanced structure and function of disordered versus folded proteome, that deviations from this ideal carry dire consequences on protein homeostasis conducive to disease protein aggregation and stress vulnerability.

## Graphical abstract

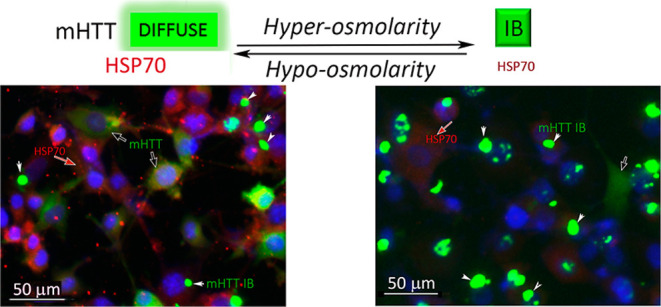

## Introduction

Aging is well-known to be associated with a multitude of primary and downstream changes in cell environment and physiology.^1^ Two remarkably common and conserved age-related changes, from yeast, worm, fish, to man, are (1) a decrease in cell hydration and (2) an increase in the propensity of a variety of proteins to desolvate into forming aggregates or precipitates in the aging cell and organism.^2–15^ Indeed, there is a large body of evidence that decreased cell hydration and slowed water mobility, as in aging cells and organisms, have knock-on effects on the mobility and function of macromolecules, and in particular, of the class of structurally expansive and dynamic intrinsically disordered proteins (IDPs) that include many regulatory and disease proteins.^9,16–27^

Huntington disease (HD) is a monogenic, autosomal dominant neurodegenerative disease (ND) caused by expansion of the polymorphic CAG trinucleotide repeat of Huntingtin gene (*Htt*) that codes for an expanded polyglutamine track of the mutant Huntingtin protein (mHTT). Symptomatic presentation of the HD trait is age-delayed and inversely correlated with the degree of polyQ expansion (>35), typically manifesting in adult disease subjects in their 40s.^28–30^ Importantly, age is well-known as the biggest risk factor for the two most common ND–Alzheimer disease (AD) with an average age of onset in the mid-60s, Parkinson disease (PD) that typically manifests between ages of 50 and 65.^31^ A shared pathological finding of these ND is the deposition of disease protein aggregates in cells/tissues known as Huntingtin inclusion bodies (IBs) in HD, β-amyloid plaque, and tau neurofibrillary tangles in AD, and Lewy body of α-synuclein aggregate in PD.^32^ The specific role(s) of disease protein aggregates, notably of Aβ amyloid plaque, in causing neuron dysfunction and death in disease states remain to be ascertain.^33–35^

The objective of this study is to gain insights into if and how age-related changes in cell hydration and macromolecular crowding can drive changes in the structural dynamics of the proteome and contribute to disease protein aggregation as well as vulnerability to environmental stresses of aging cells and organisms. For this, we use osmotic tools to effect changes in cell volume, hydration, and macromolecular crowding of a HD cell model to evaluate consequential changes in the structural dynamics of the disordered/unstructured versus the ordered/structured proteins in LIVE cells. We monitored aggregation of mHTT^Exon1^-EGFP reporter protein as a readout for the class of IDP, whereas induction of HSP70 chaperone was used to assess the dynamic instability of folded proteins upon heat stress. Our results provide evidence of a dynamic and opposing regulation of the folded versus the disordered proteome upon changes in the cell osmotic environment to underscore the critical importance of an evolutionarily conserved ∼300 mOsM iso-osmotic cell environment for proteome homeostasis and cell function.

## Results

A PC12-derived cell line—the 14A2.6 lineage—with genome-integrated ecdysone receptor-based inducible expression of the HTT103Q^Exon1^-EGFP reporter protein (from here on, abbreviated as mHTT-EGFP reporter)—is used as the HD cell model for this work.^36–39^ In our previous work, we show that experimental conditions that promote protein structuring or constrain mobility—through the induction of HSP chaperones,^37^ a transient lowering of cell incubation temperature,^39^ or the addition of stabilizing osmolytes^38^—effectively drove the compaction and aggregation of the structurally dynamic and diffusible forms of the polyQ-expanded mHTT-EGFP reporter protein into forming micron size aggregates termed “inclusion body” (IB) in live cells.^37–39^ This current work uses osmotic tools to probe if and how changes in the cell osmotic environment can tune the dynamic balance of proteins at two ends of the structural spectrum: the intrinsically disordered versus the structured and stably folded.

### Alkali-Metal Salts for Cell Media Hyperosmolarity Alter mHTT Dynamics in Cells

Salt-induced hyperosmotic stress can rapidly (within seconds to minutes) and significantly trigger hypo-hydration and macromolecular crowding in live cells.^40^ In experiments represented in Figure 1, specified concentrations (milli-osmol/liter; milli-Osmolar; mOsM) of the alkali-metal salts (LiCl, sodium chloride (NaCl), KCl and RbCl) were added to the ∼300 mOsM iso-osmotic cell medium and incubated at 37 °C for 24 h for intracellular hyper-osmolarity/hypo-hydration and to evaluate consequential changes on the dynamic balance of the diffusible versus the aggregated forms of mHTT in live cells.

**Figure 1:**
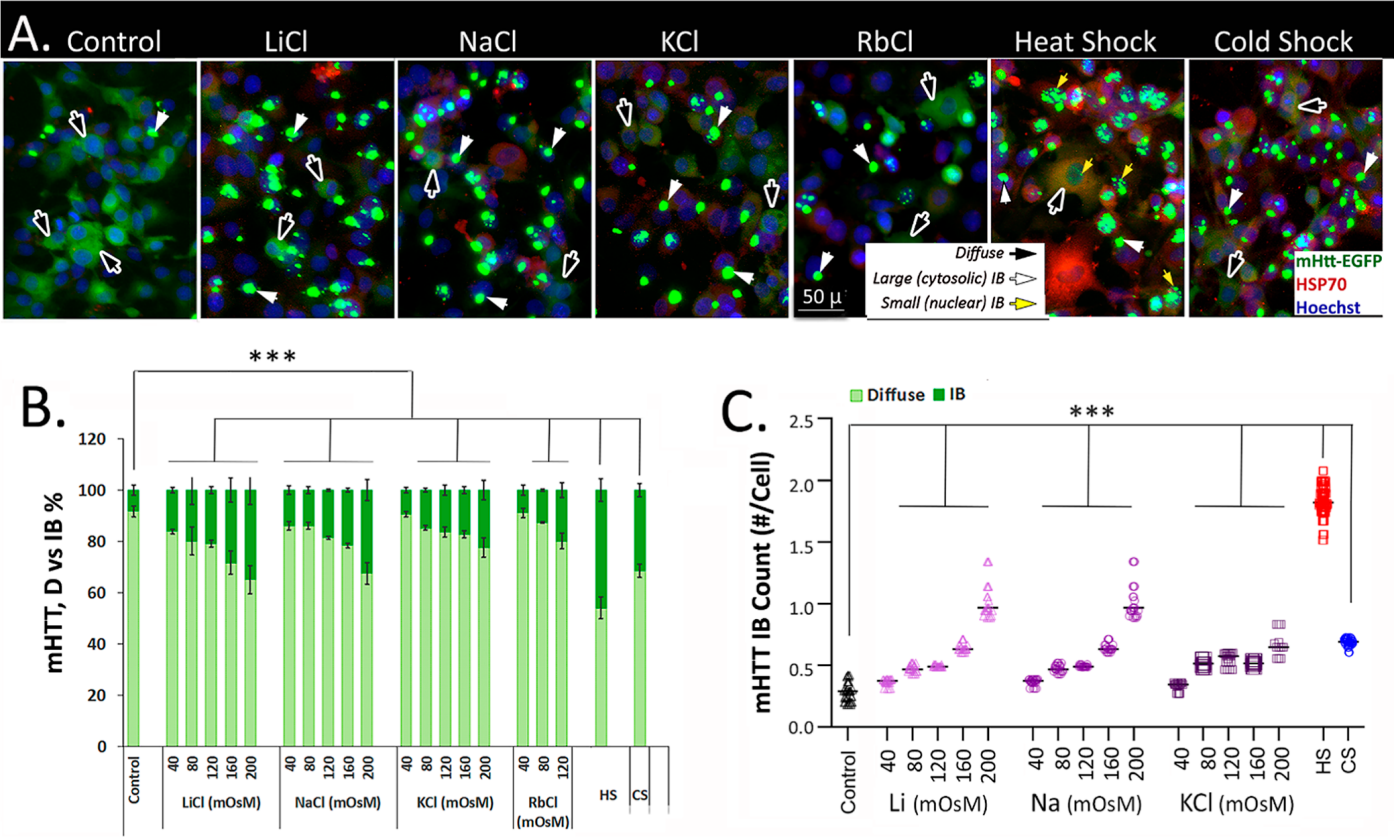
Alkali-metal salts for a hyper-osmotic cell media and macromolecular crowding in cells promoted the compaction of diffusible polyQ-expanded mHTT protein into forming micron size aggregates termed IB in a HD cell model. A PC-12 derived 14A2.6 cell line with genome-integrated ecdysone receptor-based inducible expression of the HTT^103Q^Exon1-EGFP reporter protein (abbreviated as mHTT or mHTT-reporter protein) was used for this work. Ponasterone (PA, 5 μM, 37 °C, 24 h) was added to induce the expression of the mHTT-reporter protein, and cells were plated into individual wells of a 96-stripwell plate. Alkali-metal salts—LiCl, NaCl, KCl, and RbCl—were added to designated cell wells to final concentrations as indicated (in mOsM; 10 mM = 20 mOsM) and incubated at 37 °C for 24 h prior to harvesting, fixation, and processing for immunostaining for HSP70 and Hoechst 33342 staining of cell nuclei. Images of cells that were either heat shocked at 42 °C or cold shocked at 4 °C for 2 h followed by recovery incubation at 37 °C for 22 h to promote the structuring and aggregation of the mHTT-reporter protein are included in (A) for comparison. All cell images were captured at identical settings of exposure time and light intensity/contrast. Macro programs were used for determining the diffuse versus aggregated IB mHTT intensity, mHTT IB count/cells were used to score the stacks of cell images for results shown in (B,C). (A) Representative images of control cells and cells incubated in media supplemented with 80 mM (160 mOsM) of the indicated alkali-metal salts for 24 h at 37 °C. Scale bar = 50 μm (micron). Each of the image frame shown in (A) represents an area of approximately 200 × 266 μm. Black arrows identify diffuse Htt-EGFP signal; white arrows—mHTT IB; yellow arrows—nuclear mHTT IB. (B) Percentage distribution of the mHtt signal in the diffuse versus IB format of cells incubated with the indicated mOsM of the alkali-metal salts at 37 °C for 24 h. (C) IB count/cell. Note: the significantly higher IB count/cell of the HS cells, well above other experimental conditions, is due to increase in the number of small nuclear IBs (diameter ∼ 2 μm), as previously reported. Probability of differences *P* > 0.05 is defined as not significant, between 0.01 and 0.05 is significant (*), <0.01 is very significant (**), and <0.001 is extremely significant.

Representative cell images in Figure 1A show that while >95% of the mHTT signal of control cells in iso-osmotic medium was in a soluble and diffuse format, a 24 h incubation at 37 °C of cells in media supplemented with 160 mOsM (80 mM) of alkali-metal salts—LiCl, NaCl, KCl, RbCl—effectively drove the compaction and aggregation of diffuse mHTT into forming bright, micron size (average diameter ∼ 5 μ) cytosolic IB. For comparison, images of cells that were heat shocked at 42 °C or cold shocked at 4 °C for 2 h followed by recovery incubation at 37 °C for 22 h—conditions previously shown to promote mHTT aggregation and IB formation^37,39^ —are included in the cell image panel of Figure 1A.

To assess and compare the rank order of efficacy of these salts in promoting mHTT aggregation for IB formation, we quantitate and show in Figure 1B the % of mHTT in diffuse versus aggregated IB format of cells incubated with specified concentrations of the salts at 37 °C for 24 h. mHTT IB count per cell under the specified treatment conditions is shown in Figure 1C. Results show that the addition of alkali-metal salts (LiCl, NaCl, KCl, and RbCl) to media of cells kept under the normal growth temperature of 37 °C drove mHTT IB formation in proportion to the [mOsM] of salts added. The observed rank order of efficacy of this salt-dependent effect is LiCl ≥ NaCl > KCl ≥ RbCl—a rank order that approximates the in vitro effects of these salts on α-synuclein aggregation as previously reported^26,41^ to perhaps suggest a ubiquitous effect of alkali-metal salt ions on water mobility that impinges on mobility of the structurally expansive disease protein for compaction and aggregate formation. These micrometer-size cytosolic mHTT IBs as shown in Figure 1 are end-stage, highly cross-linked material that can be sedimented by centrifugation of cell lysates; their ubiquitous round shape suggests their formation via liquid–liquid phase separation.

Immunostaining of cells for the HSP70 chaperone protein (Figure 1A, cell images, red signal)—as a readout of the lability of folded proteins—shows that alkali-metal salts had little or no effect on the low, basal HSP70 expression of cells under the normal growth temperature of 37 °C. For comparison, results show a robust increase of the HSP70 protein after a 2 h heat shock at 42 °C as well as a more modest increase in cells subjected to a 2 h cold shock at 4 °C followed by recovery incubation at 37 °C as previously reported.^37,39^

### Diametrically Opposed Regulation of mHTT Aggregation versus HSP70 of Cells under Hyper- and Hypo-Osmotic Stress

The eukaryotic proteome spans the entire structural spectrum—from the stably folded to the intrinsically disordered, with the % of disordered proteins increasing over the course of evolution.^42,43^ Teleologically, macromolecular crowding versus dispersion are expected to have opposite effects on the native states of proteins on these two ends of the structural spectrum:^44,45^ crowding drives compaction that would principally impact the class of structurally expansive and dynamic IDPs, whereas macromolecular dispersion would promote unraveling of compactly folded protein structures, particularly under destabilizing conditions such as upon a transient heat shock of the cells.

For studies represented in Figure 2, the physiological salt NaCl and water were used to titrate cell media osmolarity to effect changes in intracellular hydration and macromolecular crowding and to assess downstream effects on: (1) the compaction and aggregation of mHTT into forming IB as a readout for the class of structurally expansive and disordered proteins and (2) induction of HSP 70 chaperone after a transient heat shock as a readout of the lability of natively folded proteins under stress and their need for help from HSP chaperones to refold for function. Representative images of cells maintained in iso- (300 mOsM)-, hyper- (440 mOsM)-, and hypo- (200mOsM)-osmotic cell medium under the 37 °C control condition as well as after a 2 h heat shock at 42 °C followed by recovery incubation at 37 °C for 24 h are shown in Figure 2A. The % mHTT signal in diffuse versus aggregated IB format under specified experimental conditions is shown in Figure 2B, immuno-stained intensity of the HSP70 chaperone protein is shown in Figure 2C, and timeline of the experiment is shown in Figure 2D.

**Figure 2:**
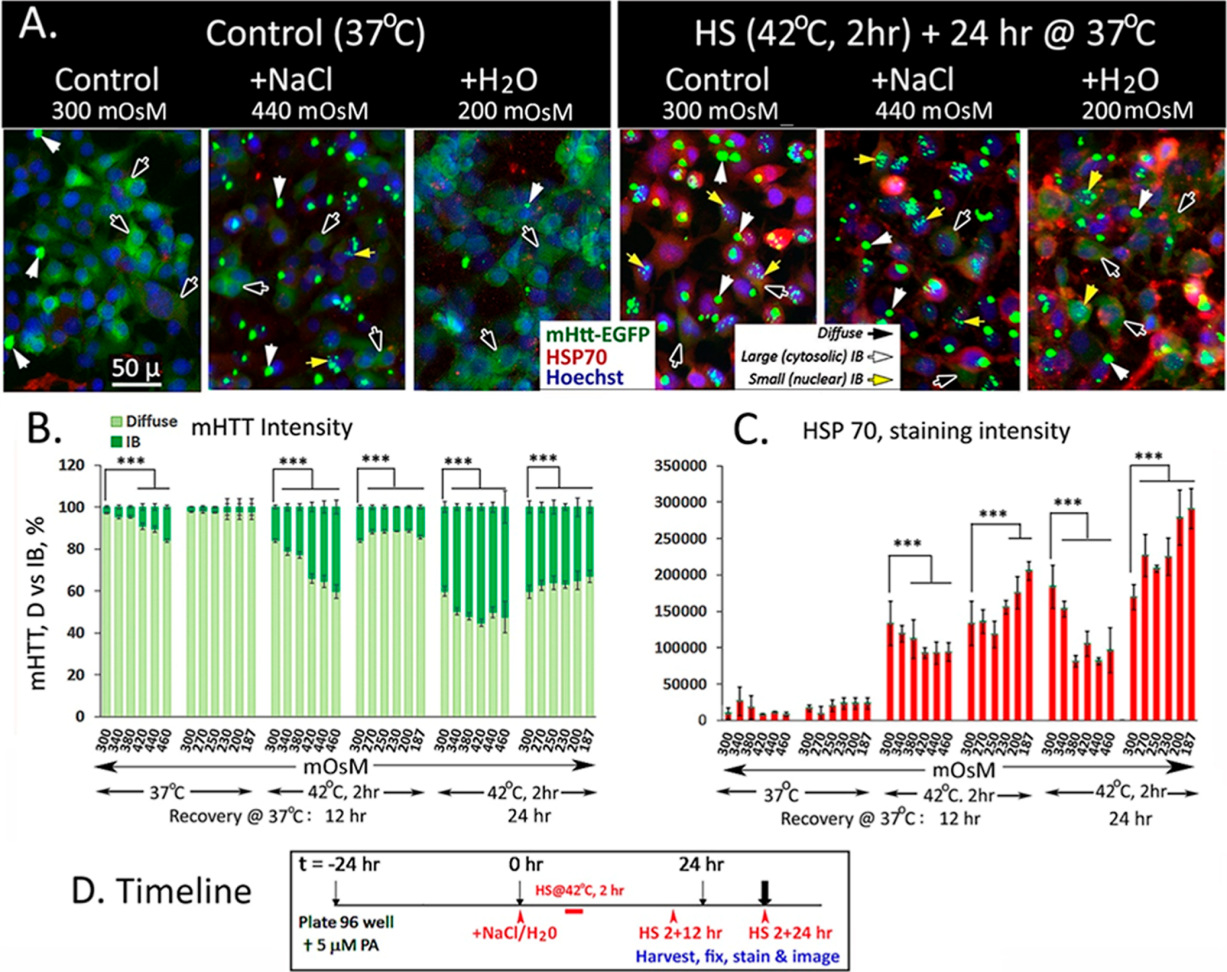
Diametrically opposed regulation of mHTT aggregation versus induction of the HSP70 chaperone protein by changes in the cell osmotic environment. Cells were plated in a 96-well plate with 5 μM ponasterone to induce the expression of the mHTT-EGFP reporter protein for 24 h at 37 °C. NaCl or water was added to individual wells of cells to final media osmolarity as indicated and equilibrated at 37 °C for 6 h. Designated stripwell of cells was heat shocked at 42 °C for 2 h followed by recovery incubation at 37 °C for either 12 or 24 h prior to harvesting for cell fixation and processing for immunostaining for HSP70 protein and cell nuclei. A parallel set of cells kept at the 37 °C temperature served as the control. (A) Representative images of cells maintained in the iso- (300 mOsM), hyper- (440 mOsM), and hypo-osmotic (200 mOsM) cell media under the normal 37 °C incubation temperature and after a transient 2 h heat shock at 42 °C followed by recovery incubation at 37 °C for 24 h. Cells were fixed and stained for HSP70 (RED) and cell nuclei (BLUE). Each of the images in Figure 1A represents an area of approximately 200 × 266 μm. Black arrows identify diffuse Htt-EGFP signal; white arrows—IB; yellow arrows—nuclear IB. Scale bar = 50 μm. (B) Percentage distribution of the mHtt signal in the diffuse versus IB format in control and heat shocked cells maintained in cell media of indicated osmolarity for time (hr) as specified. (Note: the total amount of mHTT protein per unit cell (diffuse + IB) did not change significantly as a function of treatment conditions). The tapered increase in % mHTT IB signal of HS cells maintained in the 440 and 460 mOsM media at the longer 24 h recovery incubation time 5th set of bar graphs in (B) may be attributable to (1) Csat (saturating concentration) of the mHTT protein that governs the desolvation and aggregation of mHTT into forming IB at a given [mHTT] in cells, and (2) the concurrent tapering in HSP70 induction of cells in high salt media (see result in C) thus limiting the pro-structuring effect of HSP chaperone for mHTT compaction, aggregation, and IB formation. (C) Quantitation of HSP70 signal intensity/cell under the different treatment conditions by immunostaining with a rabbit monoclonal antibody against the HSP70 protein. (D) Timeline of the experiment.

For cells maintained at the normal growth temperature of 37 °C in iso-osmotic cell media, >90% of the mHTT signal is in the soluble “diffuse” format (Figure 2A, left: 37 °C control). The addition of NaCl to increase media osmolarity (300–460 mOsM) supported a graded increase in mHTT compaction and aggregation in live cells; increasing the % of mHTT IB signal intensity from the iso-osmotic baseline of ∼3% to ∼15% for cells incubated in the 460 mOsM hyper-osmotic medium at 37 °C for 24 h (Figure 2B, 1st set of bar graphs).

Heat shock through induction of HSP chaperones has been shown to promote the structuring and compaction of diffuse mHTT into forming IB.^37^ Here, we investigated whether the heat shock-induced increase in mHTT IB formation may be titrated by concurrent changes in cell media osmolarity. Representative cell images on the right-half of Figure 2A show differences in form and intensity of the mHTT (GREEN) and HSP70 proteins (RED) of heat shocked cells maintained in iso- (300 mOsM), hyper (440 mOsM), and hypo (200 mOsM)-osmotic cell media. Quantitation of the % of mHTT signal in the diffuse versus aggregated IB format of Figure 2B shows that heat shock followed by recovery incubation at 37 °C for either 12 or 24 h increased mHTT aggregation into forming micron-sized IBs, as we previously reported.^37^ Importantly, this HS-induced increase in mHTT IB formation is strongly and proportionally enhanced by increases in cell media osmolarity from 300 to 460 mOsM and conversely, tapered by decreases in cell media osmolarity from 300 to 187 mOsM. Qualitatively similar results are observed after either a 12 or 24 h of recovery incubation at 37 °C after HS at 42 °C, with the longer 24 h recovery incubation period supporting a greater increase in mHTT aggregation and IB formation (as a % of total mHTT, Figure 2B).

Concurrent assessment of [HSP 70] by immunostaining of the cells in Figure 2C show that (1) a transient heat shock at 42 °C followed by recovery incubation at 37 °C significantly increases the abundance of HSP70 protein at both the 12 and 24 h of recovery incubation, with the increase being ∼30–40% higher after the longer 24 h than the shorter 12 h of recovery incubation at 37 °C. (2) This HS-induced HSP70 accumulation is proportionally constrained by increases in cell media osmolarity from 300 to 460 mOsM for macromolecular crowding and structural compaction. (3) Conversely, decreases in cell media osmolarity from 300 to 187 mOsM for macromolecular dispersion and relaxation proportionately boosted HSP70 induction by a transient HS of the cells (Figure 2C).

### Cell Osmotic Environment Dynamically Modulates the Heat Shock Transcriptional Response

The heat shock response (HSR) is an evolutionarily conserved, stress-induced transcriptional response initiated by temperature- or stress-induced unfolding and exposure of buried hydrophobic core of natively folded protein structures leading to activation of heat shock factor 1 (HSF1) transcription factor and increase transcription and translation of a class of protein chaperones, the heat shock proteins (HSPs).^46–48^ HSPs are crucial for homeostasis of the folded proteome under stress (aka: proteostasis): to shelter exposed hydrophobic core and prevent aggregation of unfolded protein structures, to assist in their refolding, as well as in facilitating degradation—processes that are critically important for restoration of the folded proteome for function after stress.^47^ Additionally, HSPs play important roles in cell signal transduction, cell cycle, and apoptosis regulation, and dysregulation of HSPs is implicated in various disease states.^49^ In summary, the HSR principally functions as a “guardian” of the FOLDED proteome. Accordingly, conditions that consolidate folded protein structures in cells would be expected to reduce the need for HSP chaperones, whereas conditions that destabilize folded protein structures to expose the hydrophobic core would augment the need for HSP chaperones for the restoration of the natively folded protein structure after stress.

In experiments represented in Figure 3, cells equilibrated in hypo-, iso-, and hyper-osmotic media of 200, 300, and 460 mOsM were heat shocked at 42 °C for 2 h followed by recovery incubation at 37 °C for the times indicated to determine HSP70 induction and accumulation. Figure 3A,B presents results from immuno-Western blot quantitation of the HSP70 protein to show a time-dependent increase in abundance of HSP70 protein upon recovery incubation of cells at 37 °C after a transient 2 h HS at 42 °C; the rank order of this heat shock-induced increase in HSP70 protein accumulation is hypo-osmotic (200 mOsM) > iso-osmotic (300 mOsM) ≫ hyper-osmotic cell medium (460 mOsM).

**Figure 3:**
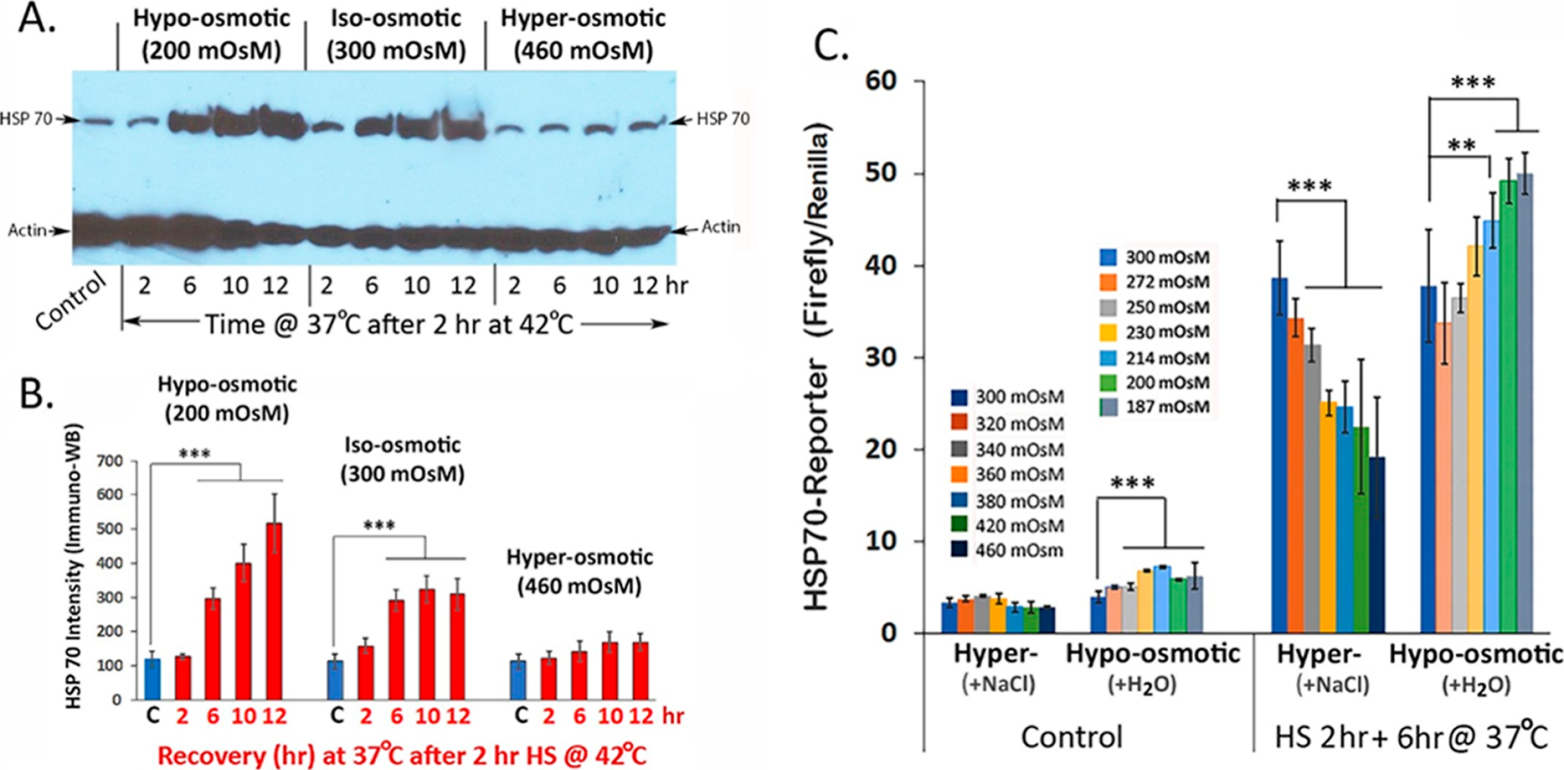
Cell osmotic environment modulates the HSR. (A) Immuno-Western blot probing for HSP70 in extracts of cells maintained in hypo- (200 mOsM), iso- (300 mOsM), and hyper-osmotic cell media for times (hr) as indicated. Water and NaCl were used to titrate media osmolarity of cells in 60 mm tissue culture and equilibrated at 37 °C for 4 h. Cells were heat shocked at 42 °C for 2 h followed by recovery incubation a 37 °C for 2, 6, 10, and 12 h. Cells were harvested and whole cell extracts prepared according to methods described. (A) Aliquots of whole cell extracts containing 20 μg of protein were processed for SDS-polyacrylamide gel electrophoresis followed by immune-Western blot detection and quantitation of the heat inducible HSP70 protein according to methods described. The positions of the HSP70 protein and the 42 kDa actin are shown as indicated. (B) Relative amounts of the HSP70 protein under specified treatment conditions (*n* = 3). (C) Quantitation of hsp70 promoter-driven luciferase reporter gene activity of cells kept in hyper- and hypo-osmotic cell media. Probability of differences *P* < 0.01 is very significant (**), and <0.001 is extremely significant.

In experiments represented in Figure 3C, we utilized a hsp70 gene promoter-driven firefly luciferase reporter to further assess the effects of changes in the cell’s osmotic environment on the hsp70 gene promoter-driven-firefly luciferase reporter to further assess the effects of changes in the cell osmotic environment on induction of the heat shock transcriptional response. For this, cells were transfected with the hsp70-luciferase reporter DNA along with the renilla luciferase (RLU) DNA as an internal control.^37^ The result shows that the heat shock-induced increase in hsp70-reporter gene activity is proportionately repressed by the stepwise increase in cell media osmolarity from 300 to 460 mOsM. Conversely, this HS-induced hsp70 promoter-reporter gene activity is proportionately boosted by the stepwise decrease in cell media osmolarity from 300 to 187 mOsM for macromolecular dispersion and unraveling of folded protein structures under stress. Assessment of the basal (i.e., 37 °C) hsp70-reporter gene activity in Figure 3C shows a small while statistically significant increase in the basal hsp70-reporter gene proportional to the decrease in cell media osmolarity from 300 to 187 mOsM, whereas increasing cell media osmolarity from 300 to 460 mOsM has a trending but not statistically significant effect on the basal 37 °C hsp70-reporter gene activity.

Together, the results in Figure 3 along with the HSP70 immunostaining result of Figure 2C provide clear evidence that induction of the heat shock transcriptional response, for restoration of the folded proteome after a transient heat stress, is proportionately titrated by changes in the cell osmotic environment: suppressed under a hyper-osmotic environment for crowding and stabilization of the folded proteome and conversely, enhanced under a hypo-osmotic environment for macromolecular dispersion and destabilization of folded protein structures upon heat stress.

### Cell Impermeant Polyethylene Glycols Promote the Compaction and Aggregation of PolyQ-Expanded mHTT to Form IB in Live Cells

To ascertain the observation of an osmolarity-dependent modulation of mHTT dynamics and to mitigate concerns due to nonspecific ionic and charge effects of the salts used, we turned to a class of noncharged osmolytes—polyethylene glycols (PEG), entities well-known for their efficacy in increasing fluid osmotic pressure to cleanse the gastrointestinal tract in clinical procedures.^50^ Our preliminary tests included PEG 400, 600, 1500, 2000, and 3000 (https://rigakureagents.com/). The lower-molecular weight PEG 400 and 600 proved to be acutely cytotoxic, most likely due to their lower-molecular weights and permeation into cells as previously reported.^51,52^ These low MW PEGs were therefore excluded from further experimentation.

Representative images of control cells and cells incubated in media supplemented with 4% PEG 1500, 2000, and 3000 at 37 °C for 8 h are shown in Figure 4A. These cell images along with results on IB count/cell in Figure 4B show that the addition of 4% PEG 2000 and 3000 to the cell media resulted in an incubation time-dependent increase in mHTT aggregation and IB formation. Under the same experimental condition, PEG 1500 had no significant effect on mHTT dynamics for up to 8 h of incubation with the cells at 37 °C. Analysis of IB size distribution of the 4% PEG2000-treated cells in Figure 4C shows notable increases of small nuclear IB with an average diameter of ∼2 μm, particularly at the longer time of treatment (6–8 h) of the cells with PEG 2000 as well as the larger cytosolic IB with an average diameter of ∼5–6 μm. This observed increase in small nuclear IB in the PEG 2000/3000-treated cells is reminiscent of the effects of a transient heat shock, as we previously reported.^37^

**Figure 4:**
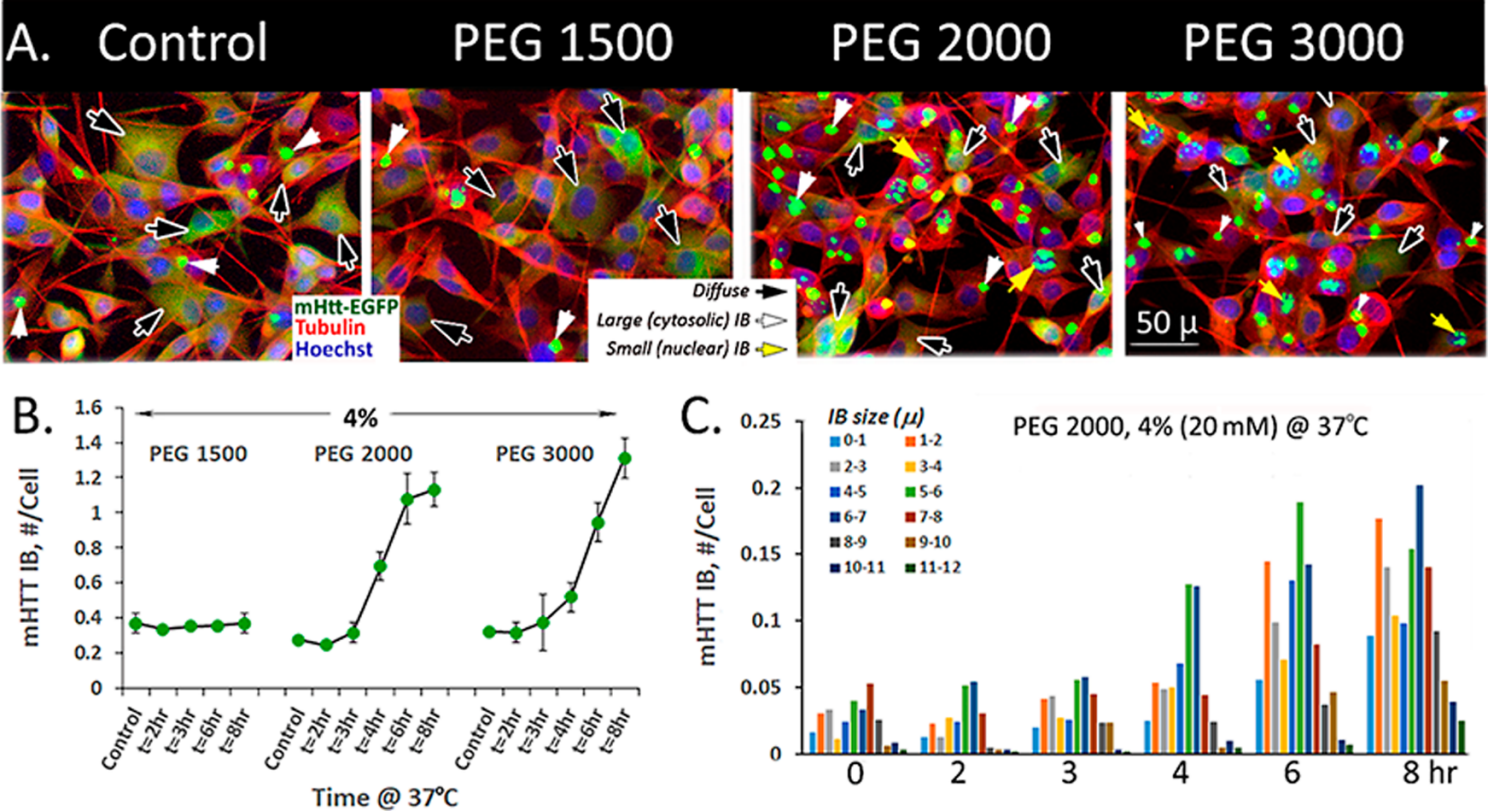
A comparison of the effects of PEG 1500, 3000, and 3000 on the compaction and aggregation of mHTT-EGFP reporter protein in cells. (A) Representative images of control cells and cells incubated with 4% PEG 1500, 2000, and 3000 at 37 °C for 8 h. Cells were fixed and immuno-stained for actin. Black arrows identify diffuse Htt-EGFP signal; white arrows—IB; yellow arrows—nuclear IB. Scale bar = 50 μm. (B) mHTT IB count per cell as a function of the indicated times of incubation with PEG 1500, 2000, and 3000 at 37 °C. (C) Size profiling of mHTT IB of cells incubated with 4% PEG2000 at 37 °C for times as indicated. Macro programs for mHTT IB counting and size profiling were used to obtain the results shown in (B,C).

### Cell Volume Assessment

Changes in the osmotic cell environment have rapid (within seconds to minutes) and dynamic effects on cell volume and macromolecular crowding.^40,53^ To assess and confirm the rapid volume changes of cells incubated in media with added NaCl or PEG, we used a Cy5-conjugated wheat germ agglutinin (WGA) (a fluorescence probe that binds to cell surface glycans) to trace the outline of cells for the determination of cell height by fluorescent microscopy. Figure 5 presents the orthogonal view of the Cy5-WGA-labeled control cells (Figure 5A) and cells incubated in media supplemented with NaCl (70 mM, 140 mOsM; Figure 5B) and PEG 3000 (4%, 13.3 mOsM; Figure 5C) for 30 min at 37 °C prior to fixation and cell surface labeling for cell height determination.

**Figure 5:**
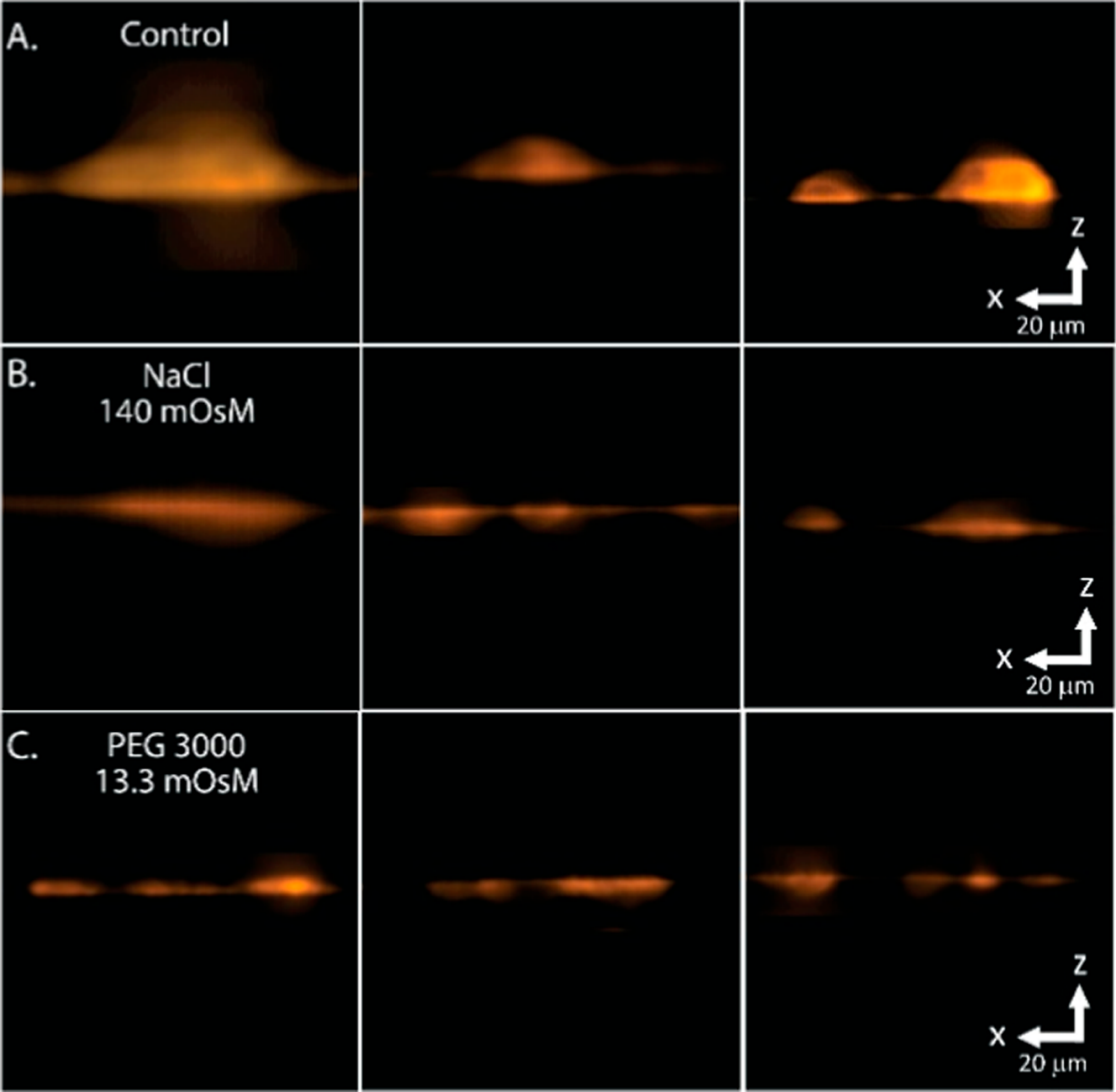
Cy5-WGA-labeled orthogonal view of cells in normal (control), versus NaCl (140 mOsM), and PEG 3000 (4%, 13.3 mOsM)-supplemented media. Cells were incubated under the specified conditions at 37 °C for 30 min prior to fixation and labeling with fluorescent WGA to mark the outline of the cell body. Confocal image stacks were acquired, and the cell profile was reconstructed from the image slices. (A) Control cells maintained in normal iso-osmotic medium, (B) cells incubated in medium supplemented with 140 mOsM (70 mM) NaCl, and (C) and medium supplemented with 4% PEG 3000 (13.3 mOsM). The orthogonal views of representative cells are presented to illustrate the thickness/height (*z*-axis) of the cell. Three independent images of cells from each condition are shown.

To quantitatively determine cell height under the different experimental conditions, the *Z*-axis fluorescence intensity profile of individual cells, namely, cell height or thickness, was determined. The full-width half-maximum (fwhm) of the fluorescent intensity profile was used as an index of cell height under the different treatment conditions. The violin plot in Figure 6A and the quantitative table in Figure 6B show that control cells had an average cell height of 25.9 μm, compared to an average cell height of 13.6 μm of the NaCl-treated cells, 20.3 μm of the PEG2000-treated cells, and 20.6 μm of the PEG3000-treated cells. These results clearly show that a 30 min incubation of cells in NaCl- or PEG2000/3000-supplemented hyperosmotic cell media will, acutely and significantly, reduce cell height and, by inference, cell volume. Assessment of cell height change upon longer incubation times in hyper-osmotic cell media (>1 h) showed a rebounded cell volume driven by the evolutionarily conserved regulated volume increase (RVI) mechanism, through adaptive increases in intracellular osmolytes and ions for osmotic equilibrium and cell volume restoration.^9^ Importantly, the intracellular environment of the newly “volume-adjusted” cell is hyper-osmotic (i.e., >300 mOsM) and in equilibrium with the hyper-osmotic extracellular environment.^9^

**Figure 6:**
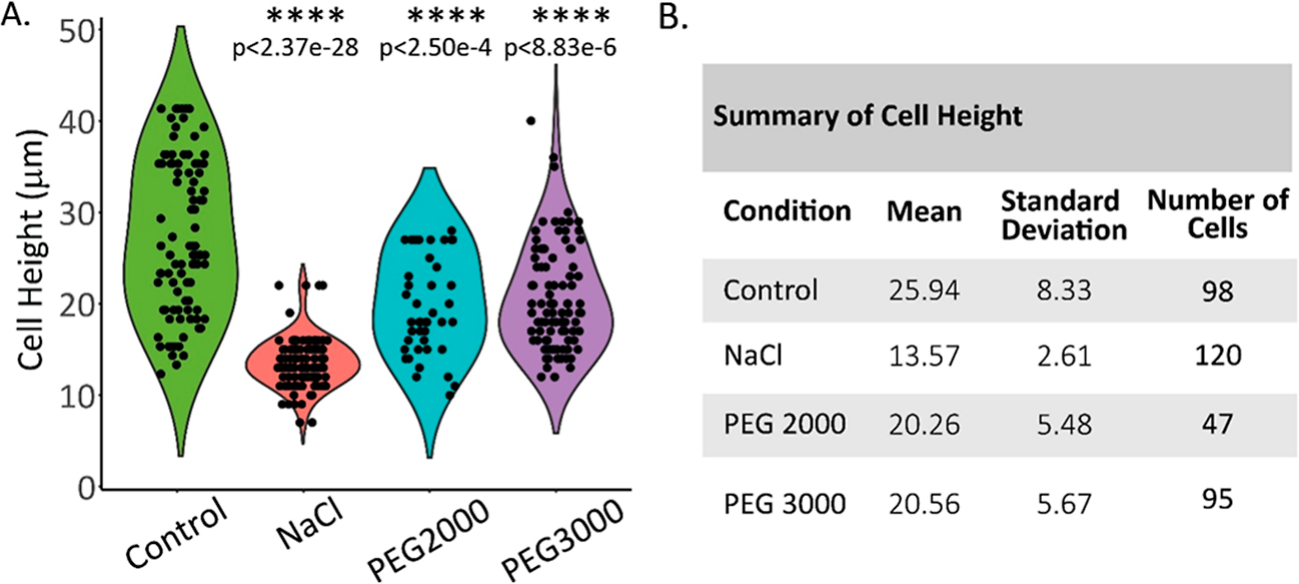
Profiling of cell size distribution of control cells, and cells incubated in media supplemented with 140 mOsM NaCl (70 mM), 20 mOsM (4%) PEG 2000, or 13.3 mOsM (4%) PEG 3000. Cells were incubated at 37 °C for 30 min prior to fixation, Cy5-WGA labeling, image acquisition, and analysis. (A) The thicknesses or heights of cells are derived from reconstructed images according to methods described in the text. Individual cell heights are shown as dots in the violin plot. *P*-value of probability of difference of the treated from that of the control indicates the differences as highly significant. (B) Summary table of mean and standard deviation of cell height of cells (μm) under the specified treatment conditions. *N* = number of cells counted.

## Discussion

“Age-related ND” underscores age as the primary risk factor for disease presentation. This is a group of diseases often characterized by widespread deposition of microscopically visible and insoluble disease protein aggregates along with the deterioration and eventual death of disease-specific neurons. The causal relationships between the widespread disease protein aggregation to result in specific neuron dysfunction culminating in disease-specific cognitive and motor dysfunction of afflicted subjects are complex and remain to be elucidated.^14,33,35,54–56^ Nonetheless, the primacy of age as the risk factor in disease manifestation, including autosomal dominant forms of the disease, suggests important age-related changes in the organism or cell environment that are conducive to disease protein aggregation and disease pathogenesis.

The focus of this work is to evaluate if and how changes in cell osmotic environments, to drive intracellular macromolecular crowding versus dispersion, can disrupt protein homeostasis for cell dysfunction and contribute to pathological changes in disease states, a hypothesis rooted in notable age-related decreases in hydration of a wide variety of cell and organismic model systems of aging research, and most importantly, of the aging human population. For example, studies on the model yeast organism have provided clear and convincing evidence of age-dependent increase in vacuole volume and a decrease in cytosolic volume, leading to macromolecular crowding and downstream physiological changes in the aging yeast cell.^2,3^ Studies on higher vertebrate animal models and human subjects have, likewise, provided unequivocal evidence of an age-related decrease in hydration for dysfunction.^4,5,7,57^ Indeed, serial NMR studies of aging humans with normal cognition over a 10 year span provided convincing and unequivocal evidence of age-related (age ∼ 40–90) acceleration of brain volume reduction, with the cortical gray matter showing a consistent pattern of volume loss in each brain lobe with aging. These changes were accompanied by increases in the CSF fluid-filled spaces.^6^

In this work, we use experimental osmotic tools to evoke changes in cell hydration for macromolecular crowding versus dispersion and to probe if and how these changes may impact the structural and functional dynamics of the proteome, from the structurally expansive and disordered to the compactly folded and ordered, to subserve the well-known observation of disease protein aggregation as well as stress vulnerability of the aging cell/organism. For this, we use a cell model of HD, whereby compaction of the structurally disordered mHTT-EGFP reporter protein into micrometer-sized aggregates termed “IB”, can be easily tracked by fluorescent microscopy and cell image analysis. Our results show that a hyper-osmotic cell medium for a crowded and hypo-hydrated intracellular environment promoted the compaction of the structurally dynamic and intrinsically disordered mHTT into forming micrometer-sized aggregates termed IB. Conversely, a hypo-osmotic medium for a hyperhydrated and dispersed intracellular environment had a more limited but nonetheless statistically significant effect in blunting mHTT compaction and IB formation. Parallel analysis of the induction of HSP70 protein in response to these changes in cell osmolarity, as a readout of the structural lability of the folded proteome under stress and their need for help from HSP chaperones to refold, revealed a pattern of regulation that is the opposite of the structurally disordered mHTT-reporter protein. We show in this work that hyperosmotic media, for hypo-hydration and macromolecular crowding, dampened, while conditions that promote intracellular hyper-hydration and macromolecular dispersion boosted the induction of HSP 70 after a transient heat shock of the cells. Remarkably, our current observation of an osmolarity-dependent suppression of HSP70 induction is reminiscent of the well-known “age”-related attenuation in induction of the heat shock transcriptional response in a wide variety of cell and organismic models of aging research as we and others have previously reported.^58–62^ Collectively, these past observations and our current results show that an attenuated heat shock transcriptional response is a characteristic feature of a hypo-hydrated and crowded cell environment that consolidates the folded proteome—be it experimentally induced as in this work, or as may naturally occur in the aging cell or organism as previously reported.^58–62^

Our proteome spans the entire structural spectrum—ranging from the stably folded to the intrinsically disordered. For higher eukaryotes, ∼30–50% of the proteome is structurally disordered either in the entirety or in parts. Folded proteins are noted for their quasi-stability, with the Δ*G* value of folded → unfolded generally falls within a relatively narrow range of 5 and 15 kcal/mol. IDPs, with their paucity of hydrophobic amino acid residues, are structurally expansive and pliable with high solvent accessibility and conformational entropy in their native states. Macromolecular crowding, through the excluded volume effect, raises the effective concentration of all solutes and limits the entropy-driven process. The impact of crowding depends strongly on the native structure of proteins under consideration—compacts the class of structurally expansive and dynamic IDPs while concurrently consolidates folded protein structures.^25,44,45,63^ At a normal growth temperature of 37 °C, crowding raises the effective concentration and constrains the dynamic mobility of IDPs to promote their compaction and aggregation, as demonstrated in this work by titrating the aggregation of the mHTT-EGFP reporter protein as a function of changes in the cell’s osmotic environment. In contrast, macromolecular crowding would limit, whereas dispersion would enhance the temperature-dependent entropic unraveling of the folded protein structures, to proportionately titrate their need and induction of HSP chaperones for refolding, as we show by the osmolarity-dependent modulation in induction of HSP70 chaperone following a transient heat shock of cells.

Importantly, there are fundamental structural and functional interplays between the “ordered” versus “disordered” proteome for the maintenance of cell homeostasis. As an example, HSF1—the key transcription factor that senses stress for induction of HSP chaperones—is equipped with structurally disordered regions necessary for function and regulation.^64^ The newly synthesized HSP chaperones also have structurally disordered regions that function as pliable molecular recognition elements to bind to exposed aggregation-prone hydrophobic patches of folded proteins to promote their refolding for restoration of cell homeostasis.^65,66^ In sum, there is a balanced dynamic interdependency in functionality of the folded versus the disordered proteome under normal iso-osmotic conditions, a balance that is perturbed by changes in the cell osmotic environment, leading to macromolecular crowding versus dispersion.

Collectively, these observations and considerations underscore the critical importance of an optimally hydrated cell environment of ∼300 mOsM, an evolutionarily conserved cell milieu across diverse organisms reflecting the critical role of cell osmolarity in macromolecular function for cell function and survival. That deviation from this ideal, as may occur in the hypo-hydrated aging and disease states,^4,5,7,57^ will have significant downstream consequences, perturbing the balanced functionality of the stably folded versus the dynamically disordered proteome and driving cell dysfunction for disease pathogenesis. As to why the brain and especially neurons may be particularly vulnerable to such age-related changes in hydration, important factors include the volume as well as ionic composition constraints that are necessary for normal function of the brain, such that the ubiquitous and effective cell volume control mechanisms employed by peripheral tissues and glial cells may have a surprisingly negative impact on neuronal functions.^9^ First, the mature brain is encased within a rigid skull, which dramatically limits the extent to which CNS tissue and its functional components can expand or shrink, and neurons are remarkable for the absence of aquaporins for water transport. Second, the brain is an excitable tissue, and changes in the intra- and extracellular levels of K^+^, Na^+^, and Cl^–^, while contributing to cell volume control in peripheral tissues, will inevitably and dramatically alter neuronal excitability. Third, hydration-related changes in the volume and function of perivascular spaces in the brain directly impact metabolic waste removal for brain homeostasis.^67^ Lastly and importantly, many of the “classic” biocompatible organic osmolytes for volume control, as in mammalian kidney and marine organisms, including glycine, gamma-aminobutyric acid, taurine, and glutamate are important signaling molecules or neurotransmitters in the CNS which preclude their use as osmo-regulators.

This work contributes to the body of literature evidence to underscore the critical importance of an iso-osmotic cell environment for balanced functionality across the cell proteome, from the compactly folded to the intrinsically and dynamically disordered. That perturbation of this balance, as may occur with aging and disease states, will have significant consequences for the functionality of the cell proteome with important downstream physiological and pathological implications.

## Methods

### Cell Culture and Treatment Conditions

The PC-12-derived 14A2.6 cell line with stably integrated DNA of the polyQ-expanded (103Q) Htt^Exon1^ sequence is as previously described.^37^ For experiments, cells were plated in 96-stripwell plates at a density of ∼0.5–1 × 10^4^ cells/well in standard Dulbecco’s minimal Essential medium. Unless indicated otherwise, experiments were generally initiated 24–36 h after ponasterone PA induction of mHtt-EGFP expression. The timeline of treatment of cells is as specified in figure legends.

Alkali-metal salts and water were used to effect changes in the cell osmotic environment and to assess the consequential changes in mHTT-EGFP structural dynamics and induction of the HSP70 chaperone protein after a transient heat shock of the cells at 42 °C. For this, salt and water were added to change the cell medium osmolarity: up from 300 to 460 mOsM for hyper-osmotic challenge or down from 300 to ∼200 mOsM for hypo-osmotic challenge. After a period of equilibration at 37 °C in the hypo- iso- or hyper-osmotic media, cells were heat shocked at 42 °C for 2 h followed by recovery incubation at 37° for time periods as indicated prior to fixation or harvesting of the cells for downstream processing.^37^ We note that under the normal 37 °C incubation temperature, cell counts and the total mHTT signal intensity per unit cell did not change significantly as a function of changes in cell media osmolarity (all within 5–10% of that of the control). A transient heat shock of the cells at 42 °C followed by recovery incubation at 37 °C lowered the total cell count, while the expression level of mHTT per viable cell (diffuse + IB) remained either unchanged or slightly higher.

For treatment of the cells with PEG, sterile PEG stock solutions were obtained from Rigaku Reagents (https://rigakureagents.com/) and diluted as necessary prior to adding to the cell media and incubation at 37 °C for the indicated time.

At the end of the experiment, cells were fixed with 4% paraformaldehyde in phosphate-buffered saline (PBS), followed by 3× wash with PBS before further processing as previously described.^37^

### Immunostaining, Image Acquisition, and Quantification

At the end of an experiment, cells were fixed with 4% paraformaldehyde in PBS followed by a 3× wash with standard PBS before further processing for immuno- and/or fluorescent-staining for β-tubulin (E7 mouse monoclonal antibody, from Developmental Studies Hybridoma Bank), HSP70 chaperone protein (ENZO Lifesciences ADI-SPA-812-D, a monoclonal rabbit antibody), or Cy5-WGA for cell surface labeling and volume assessment. Hoechst 33342 was used for staining the cell nuclei. Cell images were captured using an EVOS FL microscope equipped with a 10× objective; the field dimension of each captured image was 1200 × 900 μm (micron) with a total area of approximately 1 mm^2^. Stacks of images under specified treatment conditions were scored and analyzed using Macro programs for mHTT IB counting and size profiling as previously published.^37,38^ Each data point presented in this work is the average of 20–60 images per condition with the probability of differences clearly indicated in the figures/figure legends.

### Western Blot Detection and Quantitation of HSP70

Cells in 60 mm plates were treated under the specified experimental conditions for the indicated time periods prior to harvesting of the cells. Preparation of cell extracts, gel electrophoresis procedure, and immune-Western blot detection and quantification of the HSP70 protein were as previously described.^37^ Signal detection was performed using enhanced chemiluminescence, and HSP70 band intensity was quantitated using the ImageJ program as previously described.^37^

### Hsp70 Reporter Gene Assay

A mouse hsp70 promotor-driven firefly luciferase reporter gene was used to assess the effects of hyper- and hypo-osmotic treatment on the heat shock transcriptional response.^37^ Briefly, freshly plated 80–90% confluent cells in 35 mm or 60 mm plates were transfected with the hsp7*0*-firefly luciferase DNA, along with phRLSV40-RLU DNA, as an internal control to normalize for possible nonspecific effects of treatment conditions on reporter gene expression. The amount of each DNA used was 0.5 mg/35 mm plate or 1.5 mg/60 mm plate. The amount of lipofectamine 2000 (in μL) was 3 times that of the total amount of DNA (in μg).

6–8 h after DNA transfection, cells were subcultured and plated into individual wells of a 96-stripwell plate at a density of ∼5–6 × 10^4^ cells/well (Corning/Costar 9102) and incubated at 37 °C overnight for cell attachment and growth. To change the cell osmotic environment: (1) NaCl was added to individual cell wells for a stepwise increase in the final cell media osmolarity from 300 to 460 mOsM, and (2) water was added for a stepwise decrease in cell media osmolarity from 300 to 187 mOsM. Cells were equilibrated for 2–4 h at 37 °C. Designated stripwell of cells were then heat shocked at 42 °C for 2 h followed by recovery incubation at 37 °C for 6 h prior to harvesting and assay for reporter gene activities. A parallel set of cell samples kept under the normal 37 °C condition was used for assessment of the basal reporter gene activity.

The Dual-Glo Luciferase Assay Reagent from Promega (E2920) was used to determine first the firefly followed by the RLU activity according to the manufacturer’s instructions. Result of the Hsp70–firefly luciferase activity (in relative luminescence units, RLU) is normalized against that of the RLU as previously described.^37,38^

### Cell Size Determination—Labeling the Cell Contour with Cy5-Conjugated WGA, Image Acquisition, and Processing

Cells grown on glass coverslips were subjected to hypertonic treatment by adding NaCl or PEG3000 to the cell medium to final concentrations of 70 mM (140 mOsM) and 4% (v/v; 13.3 mOsM), respectively. Untreated cells kept in iso-osmotic cell medium served as controls. After ∼30 min of incubation at 37 °C, cells were fixed in 4% formaldehyde in 1× PBS, washed with 1× PBS, and incubated with Cy5-conjugated WGA at 4 °C overnight to label the cell membrane for downstream image acquisition and analysis. Cell images were evaluated on a custom microscope built on an Olympus IX73 instrument with a spinning disk unit (DSU) equipped with an Olympus 40× UPlanSApo NA 1.3 objective for image acquisition (Olympus Corporation, Tokyo, Japan). The Cy5 fluorescence signal was acquired by STORM, and the spinning disk in the emission light path was used to remove the out-of-focus light. Image stacks were acquired at 0.5 μm intervals to cover the entire height of the cells. Fluorescent signals were acquired at a resolution of 512 × 512 pixels on an Andor Zyla 4.2 sCMOS camera. Using the image stack, the region of interest for individual cells was defined, and the fluorescence intensity profile in the *Z*-axis (see Figure 5) for each cell was measured with ImageJ and imported into R (R project for statistical computing) for downstream analysis. In R, the *Z*-axis profile was fitted to a Gaussian curve and the baseline was determined. Cells that did not follow Gaussian fit or displayed uneven baselines were eliminated from further consideration as the measurement did not encompass the entire height/thickness of the cell. The fwhm of the curve was used as a proxy for the height/thickness of the cell (under Supporting Information, Figure S1). The Shapiro–Wilks test indicates that the data were not normally distributed, and a nonparametric Wilcoxon rank sum statistical test was employed for pairwise comparisons, as shown in Figure 6A.

### Data and Statistical Analysis

ImageJ Fiji and Macro programs were used for image analysis and quantitation essentially as previously described.^37^ Statistical analyses were done using GraphPad InStat or GraphPad Prism 6. Data shown are the mean ± SD. The significance of the difference between groups of data was determined using ANOVA followed by the post hoc Tukey–Kramer multiple comparisons test. Probability of difference *p* > 0.05 is defined as not significant, between 0.01 and 0.05 is significant (*), <0.01 is very significant (**), and <0.001 is extremely significant (***).

## Supplementary Material



## Notes

Cell height data acquisition and calculation (PDF)

## The Abbreviations Used are

Ab: beta-amyloid
AD: Alzheimer’s disease
CSF: cerebral spinal fluid
EGFP: enhanced green fluorescent protein
HD: Huntington disease
HS: heat shock
HSF: heat shock transcription factor
HSP: heat shock protein
IB: inclusion body
mHTT: poly-Q expanded mutant Huntingtin protein
ND: neurodegeneration
PD: Parkinson’s disease
PEG: polyethylene glycol
WGA: wheat germ agglutinin

## References

[1] López-Otín C., Blasco M. A., Partridge L., Serrano M., Kroemer G. (2023). Hallmarks
of aging: An expanding universe. Cell.

[2] Mouton S. N., Boersma A. J., Veenhoff L. M. (2023). A physicochemical
perspective on
cellular ageing. Trends Biochem. Sci..

[3] Mouton S. N., Thaller D. J., Crane M. M., Rempel I. L., Terpstra O. T., Steen A., Kaeberlein M., Lusk C. P., Boersma A. J., Veenhoff L. M. (2020). A physicochemical
perspective of aging from single-cell
analysis of pH, macromolecular and organellar crowding in yeast. eLife.

[4] Hooper L., Bunn D., Jimoh F. O., Fairweather-Tait S. J. (2014). Water-loss
dehydration and aging. Mech. Ageing Dev..

[5] Vashisht A., Morykwas M., Hegde A. N., Argenta L., McGee M. P. (2018). Age-dependent
changes in brain hydration and synaptic plasticity. Brain Res..

[6] Fujita S., Mori S., Onda K., Hanaoka S., Nomura Y., Nakao T., Yoshikawa T., Takao H., Hayashi N., Abe O. (2023). Characterization of
Brain Volume Changes in Aging Individuals With
Normal Cognition Using Serial Magnetic Resonance Imaging. JAMA Netw. Open.

[7] Minton A. P. (2020). Water Loss
in Aging Erythrocytes Provides a Clue to a General Mechanism of Cellular
Senescence. Biophys. J..

[8] Saarikangas J., Barral Y. (2015). Protein aggregates
are associated with replicative
aging without compromising protein quality control. eLife.

[9] Wilson C. S., Mongin A. A. (2018). Cell Volume Control
in Healthy Brain and Neuropathologies. Curr.
Top. Membr..

[10] Walther D., Kasturi P., Zheng M., Pinkert S., Vecchi G., Ciryam P., Morimoto R. I., Dobson C. M., Vendruscolo M., Mann M. (2015). Widespread
Proteome Remodeling and Aggregation in Aging
C. elegans. Cell.

[11] Harel I., Chen Y. R., Ziv I., Singh P. P., Heinzer D., Negredo P. N., Goshtchevsky U., Wang W., Astre G., Moses E. (2024). Identification
of protein aggregates in the aging vertebrate
brain with prion-like and phase-separation properties. Cell Rep..

[12] Allen M. D., Springer D. A., Burg M. B., Boehm M., Dmitrieva N. I. (2019). Suboptimal
hydration remodels metabolism, promotes degenerative diseases, and
shortens life. JCI Insight.

[13] Dmitrieva N. I., Gagarin A., Liu D., Wu C. O., Boehm M. (2023). Middle-age
high normal serum sodium as a risk factor for accelerated biological
aging, chronic diseases, and premature mortality. EBioMedicine.

[14] Karanth S., Nelson P. T., Katsumata Y., Kryscio R. J., Schmitt F. A., Fardo D. W., Cykowski M. D., Jicha G. A., Van Eldik L. J., Abner E. L. (2020). Prevalence and Clinical
Phenotype of Quadruple Misfolded
Proteins in Older Adults. JAMA Neurol..

[15] Dmitrieva N. I., Boehm M., Yancey P. H., Enhörning S. (2024). Long-term
health outcomes associated with hydration status. Nat. Rev. Nephrol..

[16] Stephens A. D., Kaminski Schierle G.
S. (2019). The role of water in
amyloid aggregation kinetics. Curr. Opin. Struct.
Biol..

[17] Salvi N., Abyzov A., Blackledge M. (2019). Solvent-dependent
segmental dynamics
in intrinsically disordered proteins. Sci. Adv..

[18] Vecchi G., Sormanni P., Mannini B., Vandelli A., Tartaglia G. G., Dobson C. M., Hartl F. U., Vendruscolo M. (2020). Proteome-wide
observation of the phenomenon of life on the edge of solubility. Proc. Natl. Acad. Sci. U. S. A..

[19] Wirth A. J., Gruebele M. (2013). Quinary protein structure and the
consequences of crowding
in living cells: leaving the test-tube behind. BioEssays.

[20] Gallat F. X., Laganowsky A., Wood K., Gabel F., van Eijck L., Wuttke J., Moulin M., Härtlein M., Eisenberg D., Colletier J. P. (2012). Dynamical coupling of
intrinsically disordered proteins and their hydration water: comparison
with folded soluble and membrane proteins. Biophys.
J..

[21] Levy Y., Onuchic J. N. (2006). Water mediation
in protein folding and molecular recognition. Annu. Rev. Biophys. Biomol. Struct..

[22] Miller C. M., Kim Y. C., Mittal J. (2016). Protein Composition
Determines the
Effect of Crowding on the Properties of Disordered Proteins. Biophys. J..

[23] Moses D., Ginell G. M., Holehouse A. S., Sukenik S. (2023). Intrinsically disordered
regions are poised to act as sensors of cellular chemistry. Trends Biochem. Sci..

[24] Moses D., Guadalupe K., Yu F., Flores E., Perez A. R., McAnelly R., Shamoon N. M., Kaur G., Cuevas-Zepeda E., Merg A. D. (2024). Structural biases in
disordered proteins are prevalent
in the cell. Nat. Struct. Mol. Biol..

[25] Zhou H. X., Rivas G., Minton A. P. (2008). Macromolecular
crowding and confinement:
biochemical, biophysical, and potential physiological consequences. Annu. Rev. Biophys..

[26] Zacharopoulou M., Seetaloo N., Ross J., Stephens A. D., Fusco G., McCoy T. M., Dai W., Mela I., Fernandez-Villegas A., Martel A. (2025). Local
Ionic Conditions Modulate the Aggregation Propensity
and Influence the Structural Polymorphism of α-Synuclein. J. Am. Chem. Soc..

[27] Hu G., Song H., Chen X., Li J. (2024). Wet Conformation of
Prion-Like Domain and Intimate Correlation of Hydration and Conformational
Fluctuations. J. Phys. Chem. Lett..

[28] Ross C. A., Aylward E. H., Wild E. J., Langbehn D. R., Long J. D., Warner J. H., Scahill R. I., Leavitt B. R., Stout J. C., Paulsen J. S. (2014). Huntington
disease: natural history, biomarkers
and prospects for therapeutics. Nat. Rev. Neurol..

[29] Finkbeiner S. (2011). Huntington’s
Disease. Cold Spring Harbor Perspect. Biol..

[30] Bates G. P., Dorsey R., Gusella J. F., Hayden M. R., Kay C., Leavitt B. R., Nance M., Ross C. A., Scahill R. I., Wetzel R. (2015). Huntington
disease. Nat. Rev.
Dis. Primers.

[31] Hou Y., Dan X., Babbar M., Wei Y., Hasselbalch S. G., Croteau D. L., Bohr V. A. (2019). Ageing as a risk factor for neurodegenerative
disease. Nat. Rev. Neurol..

[32] Ross C. A., Poirier M. A. (2004). Protein aggregation
and neurodegenerative disease. Nat. Med..

[33] Fu H., Hardy J., Duff K. E. (2018). Selective
vulnerability in neurodegenerative
diseases. Nat. Neurosci..

[34] Pandya V. A., Patani R. (2021). Region-specific vulnerability
in neurodegeneration:
lessons from normal ageing. Ageing Res. Rev..

[35] Hunter P. (2024). The controversy
around anti-amyloid antibodies for treating Alzheimer’s disease. EMBO Rep..

[36] Apostol B. L., Kazantsev A., Raffioni S., Illes K., Pallos J., Bodai L., Slepko N., Bear J. E., Gertler F. B., Hersch S. (2003). A cell-based assay for
aggregation inhibitors as therapeutics
of polyglutamine-repeat disease and validation in Drosophila. Proc. Natl. Acad. Sci. U. S. A.

[37] Chen J. Y., Parekh M., Seliman H., Bakshinskaya D., Dai W., Kwan K., Chen K. Y., Liu A. Y. C. (2018). Heat shock promotes
inclusion body formation of mutant huntingtin (mHtt) and alleviates
mHtt-induced transcription factor dysfunction. J. Biol. Chem..

[38] Aravindan S., Chen S., Choudhry H., Molfetta C., Chen K. Y., Liu A. Y. C. (2020). Osmolytes dynamically regulate mutant
Huntingtin aggregation
and CREB function in Huntington’s disease cell models. Sci. Rep..

[39] Castro
E Costa A. R., Mysore S., Paruchuri P., Chen K. Y., Liu A. Y. (2023). PolyQ-Expanded Mutant Huntingtin
Forms Inclusion Body Following Transient Cold Shock in a Two-Step
Aggregation Mechanism. ACS Chem. Neurosci..

[40] Kitamura A., Oasa S., Kawaguchi H., Osaka M., Vukojević V., Kinjo M. (2023). Increased intracellular
crowding during hyperosmotic stress. Sci. Rep..

[41] Stephens A. D., Kölbel J., Moons R., Chung C. W., Ruggiero M. T., Mahmoudi N., Shmool T. A., McCoy T. M., Nietlispach D., Routh A. F. (2023). Decreased Water Mobility Contributes To Increased
α-Synuclein Aggregation. Angew. Chem.,
Int. Ed..

[42] Uversky V. N. (2016). Dancing
Protein Clouds: The Strange Biology and Chaotic Physics of Intrinsically
Disordered Proteins. J. Biol. Chem..

[43] Uversky V. N. (2019). Intrinsically
Disordered Proteins and Their “Mysterious” (Meta)­Physics. Front. Phys..

[44] Wang Y., Sukenik S., Davis C. M., Gruebele M. (2018). Cell Volume Controls
Protein Stability and Compactness of the Unfolded State. J. Phys. Chem. B.

[45] Candotti M., Orozco M. (2016). The Differential Response of Proteins
to Macromolecular
Crowding. PLoS Comput. Biol..

[46] Lindquist S., Craig E. A. (1988). The heat-shock proteins. Annu.
Rev. Genet..

[47] Morimoto R. I. (1998). Regulation
of the heat shock transcriptional response: cross talk between a family
of heat shock factors, molecular chaperones, and negative regulators. Genes Dev..

[48] Liu, A. Y. ; Minetti, C. A. ; Remeta, D. P. ; Breslauer, K. J. ; Chen, K. Y. HSF1, Aging, and Neurodegeneration. In Cell Biology and Translational Medicine, Tissue Differentiation, Repair in Health and Disease; Turksen, K. , Ed.; Springer Nature Switzerland, 2023; Vol. 18, pp 23–49. 10.1007/5584_2022_733 35995906

[49] Hu C., Yang J., Qi Z., Wu H., Wang B., Zou F., Mei H., Liu J., Wang W., Liu Q. (2022). Heat shock
proteins: Biological functions, pathological roles, and therapeutic
opportunities. MedComm.

[50] Schiller L. R., Emmett M., Santa Ana C. A., Fordtran J. S. (1988). Osmotic effects
of polyethylene glycol. Gastroenterology.

[51] Biondi O., Motta S., Mosesso P. (2002). Low molecular
weight polyethylene
glycol induces chromosome aberrations in Chinese hamster cells cultured
in vitro. Mutagenesis.

[52] Pham
Le Khanh H., Nemes D., Rusznyák A. ´., Ujhelyi Z., Fehér P., Fenyvesi F., Váradi J., Vecsernyés M., Bácskay I. (2022). Comparative Investigation of Cellular
Effects of Polyethylene Glycol (PEG) Derivatives. Polymers.

[53] Delpire, E. ; Gagnon, K. B. Water Homeostasis and Cell Vol. Maintenance and Regulation. In Current Topics in Membranes; Levitane, I. , Delpire, E. , Rasgado-Flores, H. , Eds.; Academic Press, 2018; Chapter 1, Vol. 81, pp 3–52. 10.1016/bs.ctm.2018.08.001 PMC6457474 30243436

[54] Robinson J. L., Lee E. B., Xie S. X., Rennert L., Suh E., Bredenberg C., Caswell C., Van Deerlin V. M., Yan N., Yousef A. (2018). Neurodegenerative disease concomitant proteinopathies
are prevalent, age-related and APOE4-associated. Brain.

[55] Robinson J. L., Xie S. X., Baer D. R., Suh E., Van Deerlin V. M., Loh N. J., Irwin D. J., McMillan C. T., Wolk D. A., Chen-Plotkin A. (2023). Pathological combinations in neurodegenerative
disease are heterogeneous and disease-associated. Brain.

[56] Hardy J. A., Higgins G. A. (1992). Alzheimer’s disease: the amyloid cascade hypothesis. Science.

[57] Cowen L. E., Hodak S. P., Verbalis J. G. (2013). Age-associated abnormalities of water
homeostasis. Endocrinol. Metab. Clin. North
Am..

[58] Choi H. S., Lin Z., Li B. S., Liu A. Y. (1990). Age-dependent decrease in the heat-inducible
DNA sequence-specific binding activity in human diploid fibroblasts. J. Biol. Chem..

[59] Fargnoli J., Kunisada T., Fornace A. J., Schneider E. L., Holbrook N. J. (1990). Decreased expression of heat shock
protein 70 mRNA
and protein after heat treatment in cells of aged rats. Proc. Natl. Acad. Sci. U. S. A.

[60] Fawcett T. W., Sylvester S. L., Sarge K. D., Morimoto R. I., Holbrook N. J. (1994). Effects
of neurohormonal stress and aging on the activation of mammalian heat
shock factor 1. J. Biol. Chem..

[61] Liu A. Y., Lin Z., Choi H. S., Sorhage F., Li B. (1989). Attenuated induction
of heat shock gene expression in aging diploid fibroblasts. J. Biol. Chem..

[62] Heydari A. R., Wu B., Takahashi R., Strong R., Richardson A. (1993). Expression
of heat shock protein 70 is altered by age and diet at the level of
transcription. Mol. Cell. Biol..

[63] Gallat F. X., Laganowsky A., Wood K., Gabel F., van Eijck L., Wuttke J., Moulin M., Härtlein M., Eisenberg D., Colletier J. P. (2012). Dynamical Coupling of
Intrinsically Disordered Proteins and Their Hydration Water: Comparison
with Folded Soluble and Membrane Proteins. Biophys.
J..

[64] Westerheide S. D., Rachel R., Chase P., Bin X., Vladimir N. U. (2012). HSF Transcription
Factor Family, Heat Shock Response, and Protein Intrinsic Disorder. Curr. Protein Pept. Sci..

[65] Jaya N., Garcia V., Vierling E. (2009). Substrate binding site
flexibility
of the small heat shock protein molecular chaperones. Proc. Natl. Acad. Sci. U. S. A..

[66] Westerheide S. D., Raynes R., Powell C., Xue B., Uversky V. N. (2012). HSF transcription
factor family, heat shock response, and protein intrinsic disorder. Curr. Protein Pept. Sci..

[67] Shen T., Yue Y., Ba F., He T., Tang X., Hu X., Pu J., Huang C., Lv W., Zhang B. (2022). Diffusion
along perivascular spaces as marker for impairment of glymphatic system
in Parkinson’s disease. npj Parkinson’s
Dis..

