# Supplementary material for: Anisosmotic Modulation of Mutant Huntingtin Aggregation vis-a-vis HSP70 Induction—Implications for Aging, Hypo-Hydration, and Neurodegeneration
Source: ACS Chem Neurosci. 2026 Feb 17;17(5):1031–42. doi: 10.1021/acschemneuro.5c00966 (PMC12964416; doi:10.1021/acschemneuro.5c00966)

Supporting information for Publication

Title: Anisosmotic modulation of mutant Huntingtin aggregation vis-a-vis HSP70 induction – implications for aging, hypo-hydration and neurodegeneration

Authors: Alice Y. C. Liu[1*], Kelvin Y. Kwan[1], Clarissa Kwan[1], and Kuang Yu Chen[2]

From the [1]Department of Cell Biology and Neuroscience and [2]Department of Chemistry and Chemical Biology, Rutgers State University of New Jersey, Nelson Biology Laboratory, 604 Allison Road, Piscataway, New Jersey 08854

Fig. S1. Cell height data acquisition and calculation. **(A)** The fluorescence intensity profile from individual cells was obtained from a confocal image stack and plotted against the optical section in the image stack. Each dot represents the fluorescence intensity obtained from the indicated optical section. The blue dotted straight line at the bottom of the graph represents the empirically defined baseline, and the solid blue curve line represents the local polynomial regressive curve fit of fluorescence intensity. The full-width half mean (FWHM) was extracted from the fitted curve. The point spread function (PSF) in the Z-axis was assumed to be 1 um, so each optical section represented 1 μ (micron) in height. The width of the curve defined by the FWHM was used to represent the cell height. **(B)** Histogram of the number of cells at each cell height under the different treatment conditions. Cell heights were used to generate the violin plot shown in Fig. 6 of this work.

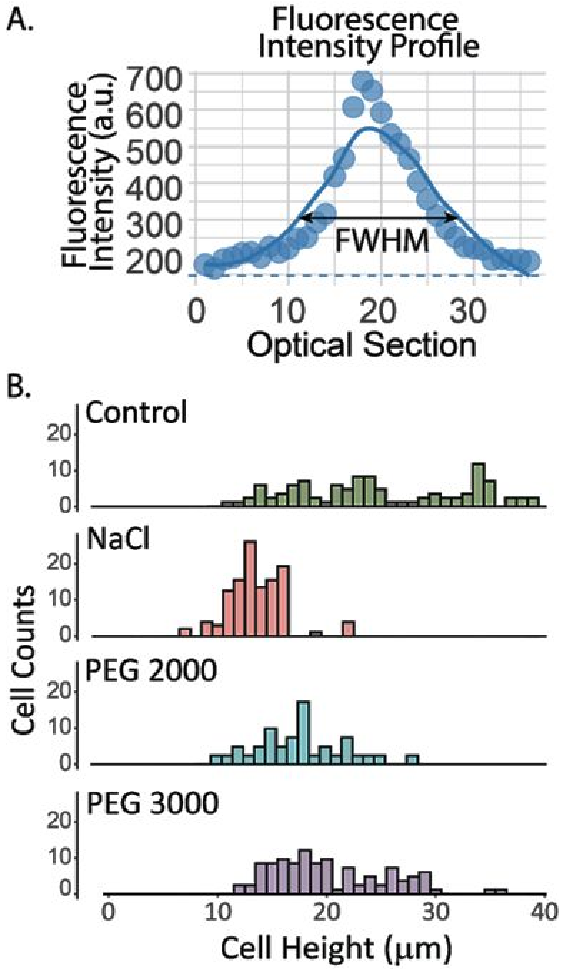

Supplement: Supplementary file 1 [file cn5c00966_si_001.pdf]
